# Anti-CCR4 monoclonal antibody KW-0761 (mogamulizumab) or investigator's choice in subjects with relapsed or refractory adult T-cell leukemia-lymphoma (ATL)

**DOI:** 10.1186/1742-4690-12-S1-P31

**Published:** 2015-08-28

**Authors:** Adrienne Phillips, Paul Fields, Olivier Hermine, Graham P Taylor, Maria Delioukina, Steven Horwitz, Juan C Ramos, Jean-Côme Meniane, Stefan K Barta, Carlos Brites, Juliana Pereria, Brady Beltran, Luis Casanova, Farooq Wandroo, Tatyana Feldman, Karen Dwyer, Michael Kurman, Kevin Conlon

**Affiliations:** 1 Weill Cornell Medical College, New York, NY, USA; 2 Guy's Hospital, London, UK; 3 Hospital Necker, Paris, France; 4 Imperial College, London, UK; 5 Cedars-Sinai Medical Center, Los Angeles, CA, USA; 6 Memorial Sloan Kettering Cancer Center, NY, USA; 7 University of Miami/Sylvester Cancer Center, Miami, FL, USA; 8 CHU de Fort de France, Fort de France, Martinique; 9 Fox Chase Cancer Center, Philadelphia, PA, USA; 10 Hospital Universitario Professor Edgard Santos-UFBA, Salvador, Brazil; 11 Hospital das Clinicas da Faculdade de Medicina da Universidade de Sao Paulo, Sao Paulo, Brazil; 12 Hospital Nacional Edgardo Rebagliati Martins, Lima, Peru; 13 Instituto Oncologico Miraflores, Lima, Peru; 14 Sandwell and West Birmingham Hospital, Birmingham, West Midlands, UK; 15 John Theurer Cancer Center at Hackensack UMC, Hackensack, NJ, USA; 16 Kyowa Hakko Kirin Pharma, Inc. Princeton, NJ, USA; 17 National Cancer Institute, Bethesda, MD, USA

## 

### Background

The receptor for macrophage derived chemokine (MDC) and thymus- and activation-regulated chemokine (TARC) CC chemokine receptor 4 (CCR4) is over expressed in several T-cell malignancies including HTLV-1 related ATL where approximately 90% of malignant cells have been shown to over express this chemokine receptor. The HTLV-1 transactivator gene (Tax) does not directly induce CCR4 expression. Rather, expression of CCR4 is controlled by the constitutive activation of several transcription factors in HTLV-1 infected cells. Inhibiting the expression of these transcription factors with small-interfering RNAs has been shown to block CCR4 expression and also reduce proliferation of the affected cells. Patients with CCR4 positive ATL are more likely to have skin involvement and shorter overall survival (OS; median 9.5 months) compared with CCR4-negative (20.6 months) patients. Mogamulizumab is a defucosylated, humanized, monoclonal antibody with enhanced antibody-dependent cellular cytotoxicity (Potelligent®) against primary ATL cells that bind to CCR4.

### Methods

This study includes patients with previously treated ATL, excluding smoldering type, who have relapsed or are refractory after at least 1 prior systemic therapy. CCR4 expression is not required for eligibility. Patients are randomized (2:1 ratio) to either mogamulizumab or Investigator's choice (pralatrexate; gemcitabine plus oxaliplatin; or dexamethasone, cisplatin and cytarabine) and stratified by disease type (ie, acute, chronic, and lymphomatous). Patients who progress on the Investigator's choice regimen may cross over to treatment with mogamulizumab. The primary endpoint is the overall response rate. Efficacy evaluation is based on the Tsukasaki criteria and includes assessment of lymph nodes, extranodal masses, the spleen, liver, skin, peripheral blood, and bone marrow. Safety analyses and an exploratory evaluation of mogamulizumab exposure-response relationship will be performed. The study is being conducted in the US, Europe, Martinique and South America. As of 19 February 2015, 67 of 70 planned patients have been randomized. Clinical Trials.gov identifier: NCT01626664.

